# Association between Lys198Asn polymorphism of endothelin‐1 gene and ischemic stroke: A meta‐analysis

**DOI:** 10.1002/brb3.1424

**Published:** 2019-09-30

**Authors:** Gaurav Nepal, Rajeev Ojha, Hari Prasad Dulal, Binod Kumar Yadav

**Affiliations:** ^1^ Maharajgunj Medical Campus Tribhuvan University Institute of Medicine Kathmandu Nepal; ^2^ Department of Neurology Maharajgunj Medical Campus Tribhuvan University Institute of Medicine Kathmandu Nepal; ^3^ Department of Biochemistry Chitwan Medical College Bharatpur Nepal; ^4^ Department of Biochemistry Maharajgunj Medical Campus Tribhuvan University Institute of Medicine Kathmandu Nepal

**Keywords:** cerebral infarction, EDN1, endothelin‐1, ischemic stroke, K198N, Lys198Asn, rs5370

## Abstract

**Background:**

Endothelin (ET)‐1 is a potent vasoconstrictor peptide produced by endothelial cells and associated with vascular dysfunction and cardiovascular disease. Lys198Asn is a single‐nucleotide polymorphism (SNP) of gene encoding ET‐1 (EDN1). It is hypothesized that it might have a role in altering ET‐1 and ultimately leading to vascular dysfunction and ischemic stroke. We therefore conducted a meta‐analysis to investigate the association between Lys198Asn polymorphism of EDN1 gene and susceptibility of ischemic stroke.

**Methods:**

This meta‐analysis was conducted according to the guidance of the Preferred Reporting Items for Systematic Reviews and Meta‐Analyses (PRISMA) statement. We searched PubMed, Google Scholar, Embase, Web of Science, J‐STAGE, and China National Knowledge Infrastructure (CNKI) for relevant studies. The association between Lys198Asn polymorphism and ischemic stroke susceptibility was evaluated by calculating the pooled ORs and 95% CIs.

**Results:**

Our analysis included 1,291 cases and 2,513 controls. Meta‐analysis established a significant association between Lys198Asn SNP of EDN1 gene and ischemic stroke when assuming either recessive model (OR: 1.30; 95% CI: 1.02–1.65; *p* = .03; *I* ^2^ = 41%) or dominant model (OR: 1.48; 95% CI: 1.24–1.76; *p* < .001; *I* ^2^ = 61%). There was no evidence of publication bias in either of the recessive model (Egger test: *p* = .23; Begg test: *p* = .85) or dominant model (Egger test: *p* = .79; Begg test: *p* = .85). A subgroup analysis based on subtypes of ischemic stroke showed that Lys198Asn SNP was only associated with large vessel infarction but not with lacunar infarction caused by small vessel disease. A subgroup analysis based on ethnicity revealed that the Lys198Asn polymorphism of the EDN1 gene was associated with ischemic stroke only in Caucasians.

**Conclusions:**

The present meta‐analysis suggests that Lys198Asn polymorphism of EDN1 gene is associated with an increased risk for ischemic stroke.

## Graphical abstract

Endothelin (ET)‐1 is a potent vasoconstrictor peptide produced by endothelial cells and associated with vascular dysfunction and cardiovascular disease. Lys198Asn is a single‐nucleotide polymorphism of gene encoding ET‐1 (EDN1). It is hypothesized that it might have a role in altering ET‐1 and ultimately leading to vascular dysfunction and ischemic stroke. We therefore conducted a meta‐analysis to investigate the association between Lys198Asn polymorphism of EDN1 and susceptibility of ischemic stroke. The present meta‐analysis suggests that Lys198Asn polymorphism of EDN1 gene is associated with an increased risk for ischemic stroke.

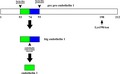

## 1 BACKGROUND

Stroke is the second leading cause of death globally and leading cause of long‐term disability in both developing and developed countries. Approximately 70% of new strokes are ischemic in origin, 51% stroke death, and 58% of stroke disability‐adjusted life years are because of ischemic stroke (Feigin et al., 2016). A complex interaction between modifiable and non‐modifiable conventional risk factors and genetic factors could be behind the pathogenesis of ischemic stroke (Boehme, Esenwa, & Elkind, 2017). Genetic influence on the pathogenesis of ischemic stroke has been established by epidemiological and animal studies (Hassan, 2000; Humphries & Morgan, 2004), and influences are polygenic whereby multiple genes exert a small influence or risk on phenotype.

The basis of ischemic stroke is the reduction in cerebral blood flow caused by alteration in structure and function of the blood vessel wall, which begins with changes in the structure and function of the endothelium (Widmer & Lerman, 2014). Endothelin (ET)‐1 is a potent vasoconstrictor peptide produced by endothelial cells and has been implicated as an important factor in the development of vascular dysfunction and cardiovascular disease (Böhm & Pernow, 2007). In addition to vasoconstriction, ET‐1 is involved in pro‐inflammatory actions, stimulation of free radical formation, platelet activation, and proliferative effects (Böhm & Pernow, 2007). Some studies have found high plasma level (Estrada et al., 2017; Sapira, Cojocaru, Lilios, & Grigorian, 2010; Ziv et al., 1992) and high cerebrospinal fluid (CSF) level (Lampl et al., 1997) of ET‐1 in ischemic stroke patients. In a study by Dai et al. (2017), injection of ET‐1 in rhesus monkey induced transient ischemic attack. Thus ET‐1 might have a role in development of ischemic stroke.

**Figure 1:**
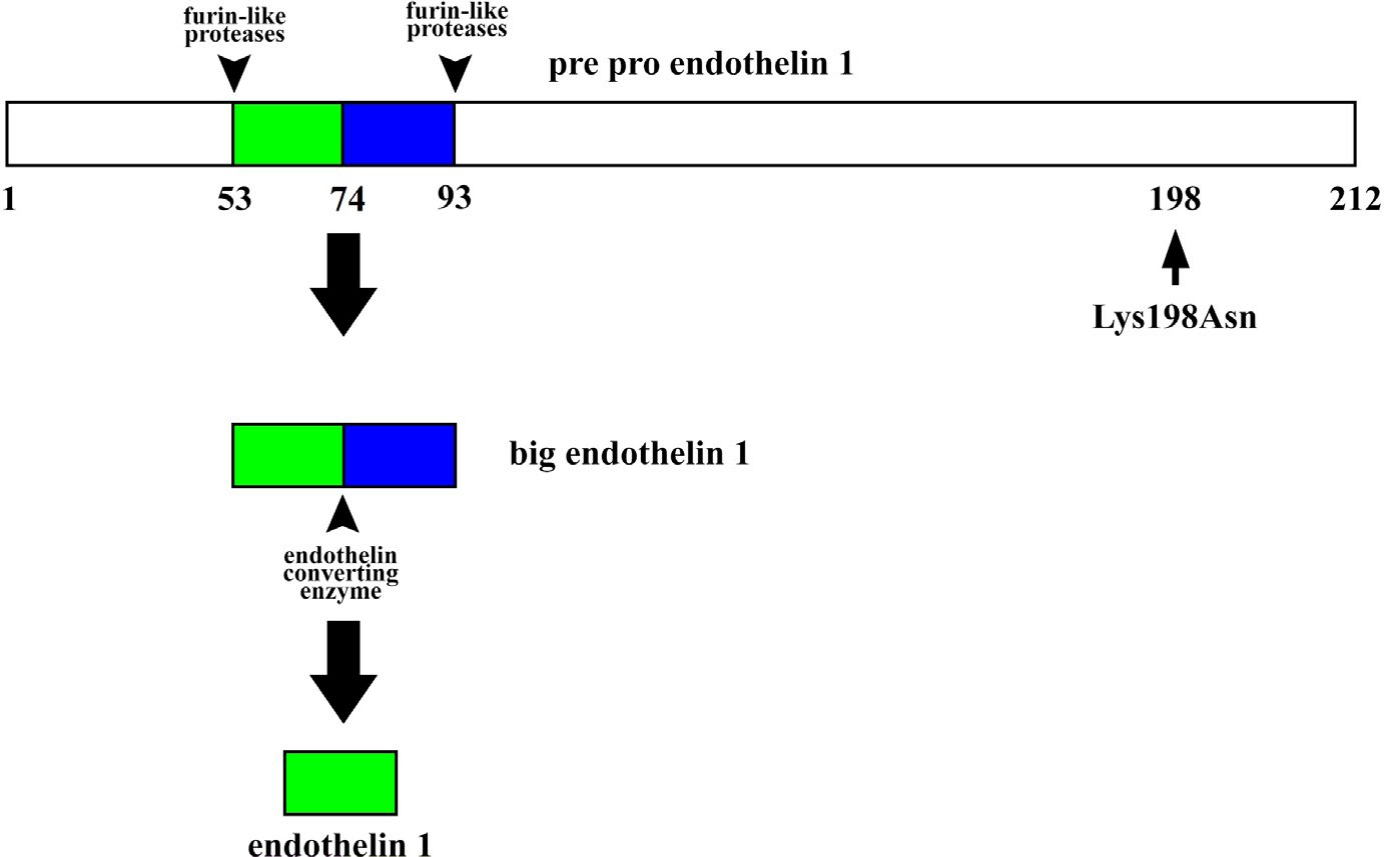
Figure showing post‐translational modification of pre‐pro‐endothelin‐1 (PPET) protein encoded by endothelin‐1 gene (EDN1). Lys198Asn polymorphism does not fall within endothelin (ET)‐1 amino acids, but is located near the carboxy‐terminal region of PPET, which is removed from PPET by furin‐like enzyme during post‐translational processing steps

The human endothelin‐1 gene (EDN1) contains 5 exons and spans ∼6.8 kb of genomic Deoxyribonucleic Acid (DNA) and is located on chromosome 6p23‐p24. Transcription of the human EDN1 gene yields a 2.8‐kb messenger Ribonucleic Acid (mRNA) that encodes a 212‐amino acid precursor protein, pre‐pro‐ET‐1 (PPET; Yanagisawa & Masaki, 1989). Furin‐like proteases cleave PPET to a 38‐amino acid protein called big ET‐1. The final cleavage step is mediated by endothelin‐converting enzymes that cleave big ET‐1 into 21‐amino acid active ET‐1 (Figure 1). K198N or Lys198Asn or rs5370 is a single‐nucleotide polymorphism (SNP) of EDN1 gene, located in its 5th exon (Tanaka, Kamide, Takiuchi, Miyata, & Kawano, 2005). This SNP leads to G‐to‐T transversion coding for a lysine (K) to asparagine (N) amino acid substitution at amino acid 198 of PPET (Tanaka et al., 2005). This SNP might have a role in altering ET‐1 and ultimately leading to vascular dysfunction and ischemic stroke. Some studies have found that Lys198Asn is associated with ischemic stroke (Dubovyk, Oleshko, Harbuzova, & Ataman, 2018; Li Wei et al., 2009; Yamaguchi et al., 2006; Zhang & Sui, 2014). However, few studies have failed to demonstrate this relationship in ischemic stroke (Aslan, Gurger, Atescelik, & Kara, 2017; Gormley, Bevan, Hassan, & Markus, 2005). Additionally, these studies are limited by their small sample size (Aslan et al., 2017; Dubovyk et al., 2018; Gormley et al., 2005; Li Wei et al., 2009; Yamaguchi et al., 2006; Zhang & Sui, 2014). Thus, we conducted a meta‐analysis to investigate the association between Lys198Asn polymorphism of ET‐1 gene (EDN1) and risk of ischemic stroke.

## 2 MATERIALS AND METHODS

This current meta‐analysis was conducted according to the guidance of the Preferred Reporting Items for Systematic Reviews and Meta‐Analyses (PRISMA) statement (Liberati et al., 2009).

### 2.1 Literature search

We searched PubMed, Google Scholar, Embase, Web of Science, J‐STAGE, and China National Knowledge Infrastructure (CNKI) for studies published from January 2000 to September 2018. Searches were conducted using the keywords “EDN1 polymorphism” or “Endothelin‐1 gene polymorphism” or “rs5370 polymorphism” or “Lys198Asn polymorphism” in combination with “stroke” or “ischemic stroke” or “cerebrovascular disease” or “cerebral infarction.” Titles and abstracts were screened and the full text of any that were considered to possibly meet the inclusion criteria were then obtained. The cited references of retrieved articles were manually checked to identify any additional eligible trials. Two authors (GN and RO) screened and retrieved reports and excluded irrelevant studies. When uncertainty about eligibility criteria arose, it was solved by discussion with all the authors.

### 2.2 Eligibility criteria

We included studies that were conducted on human subjects, and the studies were searched without any limitations on language. In this meta‐analysis, all included studies met the following criteria: (a) case–control studies focused on the association between rs5370/Lys198Asn polymorphism and ischemic stroke susceptibility, (b) patients with ischemic stroke were diagnosed with neuroimaging confirmed by neurologist, and (c) there were sufficient data of the genotypes in the case–control groups to evaluate the ORs and 95% CIs. The exclusion criteria were as follows: (a) publications with overlapping cases and controls from the similar study, (b) no genotypic data available, and (c) publications with patients with cardioembolic stroke or hemorrhagic stroke.

### 2.3 Data abstraction and assessment of methodological quality

The relevant data from each study were independently extracted by two reviewers (GN and RO) using a standardized, structured form. Extracted data included the first author, type of design, site of study, patient ethnicity, year of publication, sample size of cases and controls, control design, Hardy–Weinberg equilibrium, source of DNA, genotyping method, data of the genotypes in the case–control groups, type of ischemic stroke, mean age of participants, mean BMI of participants, and male to female ratio.

The methodological quality of each study was assessed independently by two reviewers (GN and RO) using the parameters previously used by Kumar et al. (2016) In this scale, following sections were included for assessment of methodological quality: representativeness of cases, source of controls, matching of controls, ascertainment of cases, ascertainment of controls, blinding while genotyping, genotyping methods, Hardy–Weinberg equilibrium, and association assessment. Maximum score of 2 can be provided in each section, except source of controls (maximum 3), ascertainment of controls (maximum 1), blinding while genotyping (maximum 1), and association assessment (maximum 1). Methodological quality of scale score varies from 0 to 16, in which higher score represents the better quality and lower score represents the lower quality. Any discrepancies during data extraction and quality assessment were resolved by discussion with all the authors.

### 2.4 Statistical analysis

In the majority of selected studies, the authors presented odds ratio (OR) and 95% confidence intervals (CIs) for association between Lys198Asn polymorphism and ischemic stroke susceptibility. In study where OR and 95% CI was not available, we calculated the OR and 95% CI comparing the number of genotypes in cases and controls. The association between Lys198Asn polymorphism and ischemic stroke susceptibility was evaluated by calculating the pooled ORs and 95% CIs. Heterogeneity between the included studies was determined using Cochran's *Q* test and *I* ^2^ test. The presence of *p* values above 0.1 or *I* ^2^ more than 50% was considered as an indicator of significant heterogeneity. When there was no heterogeneity, we selected the fixed effects model and, in the presence of significant heterogeneity, we selected the random effects model to calculate the effect size. Genotypes at each locus were coded as follows: TT = mutant homozygotes, TG = heterozygotes, and GG = wild homozygotes. Two genetic models were examined, including dominant model (TT + TG vs. GG), and recessive model (TT vs. GG + TG). Sensitivity analysis was performed to examine the stability of analysis. Subgroup analysis was performed based on ethnicity and stroke subtype. Meta‐regression was performed to determine whether methodological quality of study have role in effect size associated with Lys198Asn polymorphism. Egger's linear regression test and Begg's test were used for the identification of publication biases. Statistical analysis was performed using comprehensive meta‐analysis software (CMA 3.3; Biostat, 2014).

## 3 RESULTS

### 3.1 Literature search

**Figure 2:**
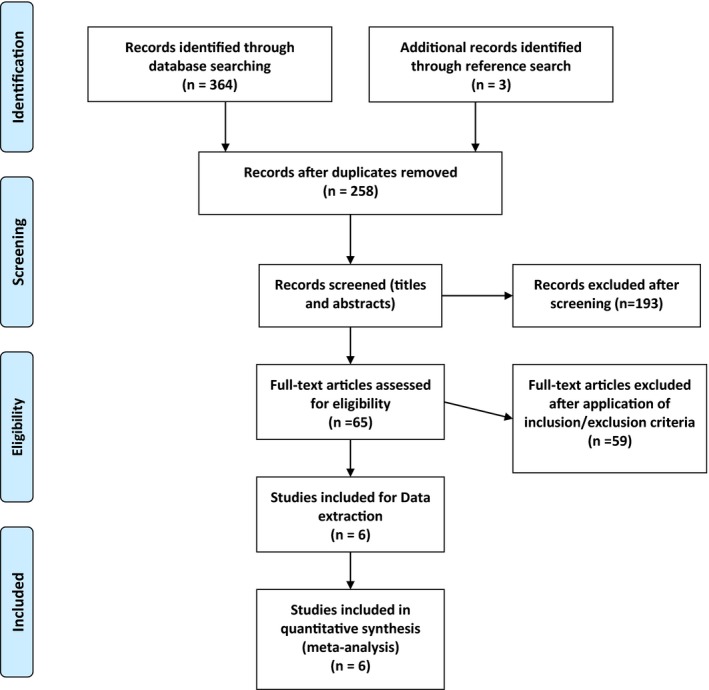
Flow of systematic literature search and selection

The results of the systematic literature search and selection are summarized in Figure 2. We identified 364 articles through database searching and three through reference lists searching. After exclusion of duplicates, 258 articles remained, and after screening titles and abstracts, 65 relevant abstracts remained. After application of inclusion and exclusion criteria, six studies were chosen for final analysis and the data were extracted.

### 3.2 Study and patient characteristics

Our research included studies from two continents and five different countries. Two studies were from China (Li Wei et al., 2009; Zhang & Sui, 2014) and one study was from Japan (Yamaguchi et al., 2006), the United Kingdom (Gormley et al., 2005), Turkey (Aslan et al., 2017), and Ukraine (Dubovyk et al., 2018) each. The publication date ranged from 2005 (Gormley et al., 2005) to 2018 (Dubovyk et al., 2018). All included studies had case–control designs. In all included studies, Hardy–Weinberg equilibrium was achieved in the control population. Venous blood was the source of DNA in all studies. Each study used a unique form of genotyping. Of the six studies, only three studies matched age and sex for controls (Gormley et al., 2005; Li Wei et al., 2009; Zhang & Sui, 2014). Four studies did inpatient recruitment of patient (Aslan et al., 2017; Dubovyk et al., 2018; Li Wei et al., 2009; Zhang & Sui, 2014) while outpatient recruitment (Yamaguchi et al., 2006) and community recruitment (Gormley et al., 2005) was done by one study each. According to quality assessment tools, the methodological quality of most studies was found to be moderately high. Table 1 lists the details of the study characteristics. The patients included in the three studies were Asian (Li Wei et al., 2009; Yamaguchi et al., 2006; Zhang & Sui, 2014) and in the remaining three were Caucasian (Aslan et al., 2017; Dubovyk et al., 2018; Gormley et al., 2005). Two studies recruited small vessel disease patients and four recruited large vessel disease patients. In the included studies, the controls had no history of cerebrovascular, coronary and other atherosclerotic diseases. Among the cases, the mean age ranged between 60 and 71 years. Among the controls, the mean age ranged between 60 and 68 years. All other patient characteristics can be seen in Table 2.

**Table 1:** Key methodological characteristics of included studies

Study	Publication date	Country	Study design	Case/Control	Control matching criteria	HWE confirmation	Source of control	DNA source	Genotyping method	Methodological quality score
Gormley et al.	2005	UK	Case–control	300/600	Age, sex	Yes	CB	Venous blood	Allele‐specific PCR	12
Yamaguchi et al.	2006	Japan	Case–control	264/1197	Not matched	Yes	OB	Venous blood	PCR and oligonucleotide probes	12
Hang et al.	2009	China	Case–control	46/50	Age, sex	Yes	HB	Venous blood	PCR‐RFLP	11
Zhang et al.	2014	China	Case–control	381/366	Age, sex	Yes	HB	Venous blood	SNaPshot genotyping	11
Aslan et al.	2017	Turkey	Case–control	100/100	Not matched	Yes	HB	Venous blood	Real‐Time PCR	11
Dubovyk et al.	2018	Ukraine	Case–control	200/200	Not matched	Yes	HB	Venous blood	PCR‐RFLP	12

Abbreviations: CB, Community based; HB, Hospital based; HWE, Hardy–Weinberg equilibrium; OB, Outpatient based; PCR‐RFLP, Polymerase chain reaction–restriction fragment length polymorphism.

**Table 2:** Patient characteristics of studies included in this meta‐analysis

Study	Ethnicity	Ischemic stroke type	Case			Control		
			Mean ± *SD* Age	Mean ± *SD* BMI	Male/Female	Mean ± *SD* Age	Mean ± *SD* BMI	Male/Female
Gormley et al.	Caucasian	Small vessel disease	67.10 ±10.26	N/A	198/102	66.85 ± 8.15	N/A	387/213
Yamaguchi et al.	Asian	Large vessel disease	68.6 ± 12.2	23.7 ± 3.5	All female	62.8 ± 12.0	23.5 ± 3.2	All female
Hang et al.	Asian	Small vessel disease	60.85 ± 13.28	Weight(kg): 66.48 ± 9.98 Height(cm): 158.35 ± 6.92	17/29	60.72 ± 11.70	Weight(kg): 63.32 ± 10.15 Height(cm): 159.60 ± 6.79	15/35
Zhang et al.	Asian	Large vessel disease	62.22 ± 10.81	24.27 ± 3.90	236/145	61.47 ± 9.64	24.34 ± 3.18	225/141
Aslan et al.	Caucasian	Large vessel disease	71.7 ± 10.6	N/A	54/66	65.8 ± 7.3	N/A	67/33
Dubovyk et al.	Caucasian	Large vessel disease	66.7 ± 10.1	27.9 ± 3.7	111/89	68.1 ± 13.9	27.3 ± 4.6	125/75

Abbreviations: BMI, Body mass index; N/A, Not available; *SD*, Standard deviation.

### 3.3 Meta‐analysis

**Figure 3:**
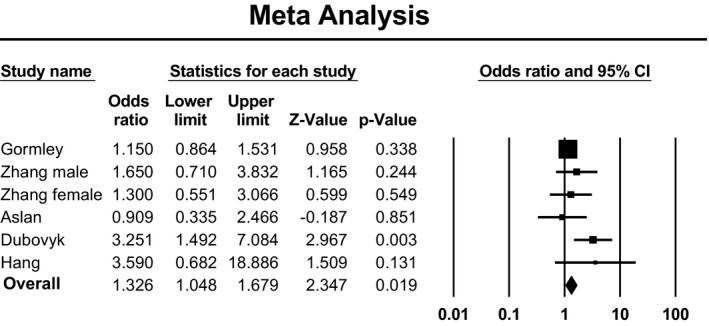
Forest plot of the result for recessive model

**Figure 4:**
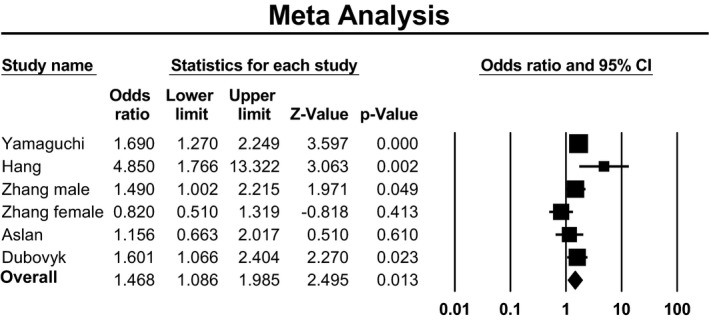
Forest plot of the result for dominant model

**Figure 5:**
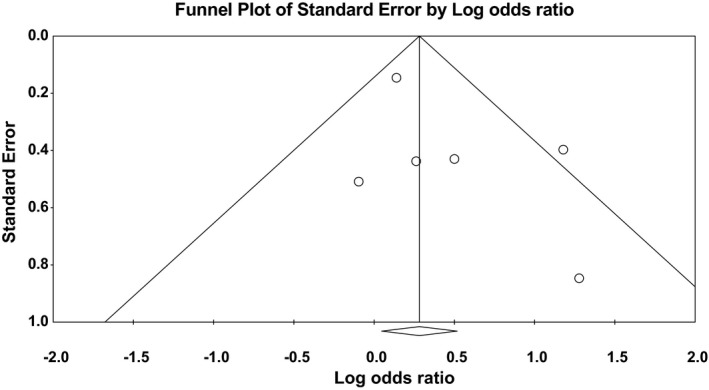
Funnel plot for detection of publication bias in recessive model

**Figure 6:**
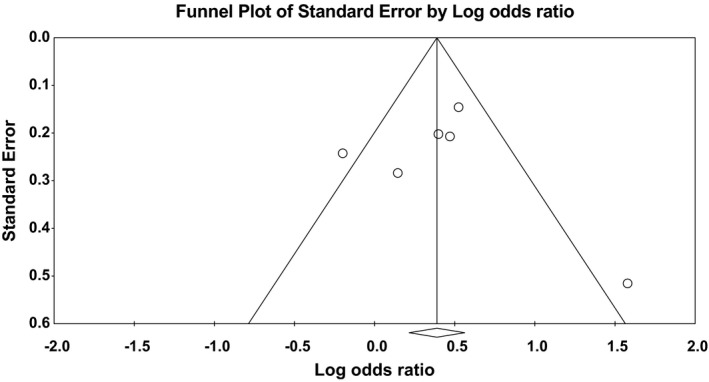
Funnel plot for detection of publication bias in dominant model

In dominant model, there was significant heterogeneity, so we applied random‐effects model to estimate pooled ORs and 95% CIs. In recessive model, there was no heterogeneity, so we applied fixed‐effects model. Meta‐analysis established a significant association between Lys198Asn polymorphism of EDN1 gene and ischemic stroke when assuming either recessive model of inheritance (OR: 1.30; 95% CI: 1.02–1.65; *p* = .03; *I* ^2^ = 41%) or dominant model of inheritance (OR: 1.48; 95% CI: 1.24–1.76; *p* < .001; *I* ^2^ = 61%). Forest plot of the result for recessive model demonstrated in Figure 3. Forest plot of the result for dominant model is demonstrated in Figure 4. There was no evidence of publication bias in either of the recessive model (Egger test: *p* = .23; Begg test: *p* = .85) or dominant model (Egger test: *p* = .79; Begg test: *p* = .85). Funnel plot for detection of publication bias in recessive model and dominant model is demonstrated in Figures 5 and 6, respectively.

### 3.4 **Sensitivity test, subgroup analysis, and meta**‐**regression**

For both models of inheritance, we sequentially excluded one study from the pooled analysis and recalculated the summary ORs to check whether the summary ORs were significantly changed. The recalculated ORs were adamant, indicating the stability of analysis.

In the recessive model, a subgroup analysis based on subtypes of ischemic stroke showed that the Lys198Asn SNP of the EDN1 gene was not associated with any of the subtypes of ischemic stroke. However, the dominant model showed that Lys198Asn SNP was associated with large vessel disease. In the recessive model, ethnicity‐based subgroup analysis showed that the Lys198Asn SNP of the EDN1 gene was not associated with ischemic stroke in both Caucasians and Asians. However, the dominant model showed that the Lys198Asn SNP was associated with ischemic stroke in Caucasians. Subgroup analysis is tabulated in Table 3.

**Table 3:** Subgroup analysis based on ethnicity and ischemic stroke subtype

Subgroups	Recessive model	Dominant model
Caucasians		
No of studies	3	2
Effect size	OR: 1.48; 95% CI: 0.74–2.97; *p* = .26	OR: 1.43; 95% CI: 1.03–1.98; *p* = .03
Heterogeneity	*I* ^2^ = 69.1%	*I* ^2^ = 0%
Asians		
No of studies	3	4
Effect size	OR: 1.62; 95% CI: 0.92–2.86; *p* = .09	OR: 1.55; 95% CI: 0.97–2.46; *p* = .06
Heterogeneity	*I* ^2^ = 0%	*I* ^2^ = 75%
Large vessel disease		
No of studies	4	5
Effect size	OR: 1.66; 95% CI: 0.98–2.8; *p* = .05	OR: 1.36; 95% CI: 1.06–1.75; *p* = .01
Heterogeneity	*I* ^2^ = 34%	*I* ^2^ = 46%
Small vessel disease		
No of studies	2	1
Effect size	OR: 1.49; 95% CI: 0.58–3.83; *p* = .41	OR: 4.85; 95% CI: 1.76–13.32; *p* = .26
Heterogeneity	*I* ^2^ = 42.9%	*I* ^2^ = 0%

**Figure 7:**
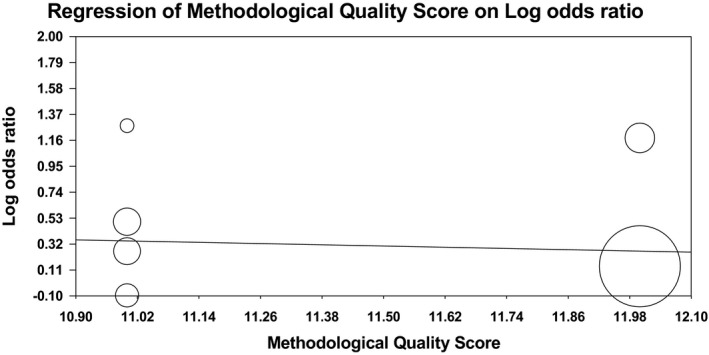
Meta‐regression plot for recessive model

**Figure 8:**
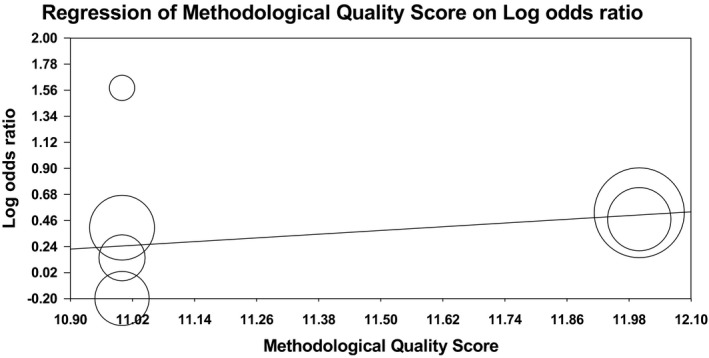
Meta‐regression plot for dominant model

Meta‐regression analysis was used to explore whether the methodological quality scores of the included studies play an important role in the association between the Lys198Asn polymorphism of the EDN1 gene and the risk of ischemic stroke. In recessive model, we found that quality scores were not significant predictors of the effect size for the association between Lys198Asn polymorphism and ischemic stroke risk (*p* = .14). However, in the dominant model, quality scores were significant predictors of the effect size (*p* = .02). Meta‐regression plot for recessive model and dominant model is shown in Figures 7 and 8, respectively.

## 4 DISCUSSION

There is growing evidence that genetic factors are involved in the development of ischemic stroke. This meta‐analysis was designed to determine an accurate estimate of the association between the EDN1 Lys198Asn SNP and the risk of ischemic stroke. Several studies have shown an independent association of Lys198Asn SNP of the EDN1 gene and number of diseases such as hypertension (Barath et al., 2007; Barbeau et al., 2003; Fang, Li, & Ma, 2017; Jin et al., 2003), obesity (Karita & Sadewa, 2019), obstructive sleep apnea (Diefenbach et al., 2009), heart failure (Colombo, Ciofini, Paradossi, Bevilacqua, & Biagini, 2006), diabetes (Li et al., 2008), pulmonary hypertension (Benza et al., 2015), intraventricular hemorrhage (SzpechtOctober , Gadzinowski, Seremak‐Mrozikiewicz, Kurzawińska, & Szymankiewicz, 2016), and subarachnoid hemorrhage (Foreman et al., 2017; Gallek et al., 2013). Therefore, the Lys198Asn SNP is considered to be a risk factor for cardiovascular disease and cerebrovascular disease. Our meta‐analysis suggests a significant association of Lys198Asn SNP of EDN1 with ischemic stroke. Our findings are consistent with the results of most of the included studies.

Lys198Asn SNP does not belong to the nucleotide sequence encoding the ET‐1 amino acid, but is located near the nucleotide sequence encoding the amino acid adjacent to the carboxy‐terminal region of PPET (Figure 1), which is removed from PPET by furin‐like enzyme during post‐translational processing steps (Tanaka et al., 2005). Considering the location of this SNP, it is unlikely that structural changes will be made in ET‐1. However, variation in gene regulation is more important in disease causality than in structural changes in specific proteins. Although Lys198Asn does not structurally alter the ET‐1 protein, studies have demonstrated that silent SNP like Lys198Asn can affect human mRNA synthesis, splicing, processing, stability, transport, translation, and protein folding (Robert & Pelletier, 2018; Shen & Basilion, 1999). Furthermore, Lys198Asn SNP can also be coinherited together with another gene highly associated with ischemic stroke due to physical proximity or with a highly functional SNP within the EDN1 gene itself, for the same reasons as above, and can structurally alter ET‐1 (Tanaka et al., 2005). In other words, above‐mentioned SNP and the causative genetic factor might be in linkage disequilibrium (Tanaka et al., 2005). Such SNP may not have influence on disease susceptibility however they can be an important maker if they coinherit with other highly disease‐associated gene or with other highly functional SNP of same gene.

In our subgroup analysis, we found that Lys198Asn SNP was only associated with large vessel infarction but not with lacunar infarction caused by small vessel disease (SVD). This may be due to a number of issues, including sample size, heterogeneous pathophysiology, and silent SVD. Silent SVD is defined as hyperintensities of the white matter that correlate with an asymptomatic cerebral infarction. Many cases of lacunar infarctions due to SVD are silent. As these cases often do not present to the hospital, they are excluded from the analysis. Complex interactions between genetic and nongenetic factors have been reported to play an important role in the progression of ischemic stroke (Boehme et al., 2017). Ethnicity is one of the important nongenetic factors. Therefore, we conducted an ethnicity‐based subgroup analysis. In the recessive model, the Lys198Asn SNP of the EDN1 gene was not associated with an ischemic stroke in both Asians and Caucasians. However, in dominant model, the Lys198Asn SNP of the EDN1 gene was associated with ischemic stroke in Caucasians but not in Asians. In Asians, especially in Chinese, lacunar infarctions due to SVD are believed to be responsible for a higher proportion of ischemic stroke than in Caucasians (Tsai, Thomas, & Sudlow, 2013). Since the Lys198Asn polymorphism is not associated with lacunar infarction due to SVD, this could be the reason why the Lys198Asn SNP of the EDN1 gene is not associated with an ischemic stroke in Asians. Yamaguchi et al. (2006) included female patients only for analysis of Lys198Asn SNP and it was significantly associated with ischemic stroke. Dubovyk et al. (2018) and Zhang and Sui (2014) calculated separate estimate for male and female and Lys198Asn SNP was significantly associated with ischemic stroke in both sex. Aslan et al., Gormley et al., and Hang et al. did not perform sex stratified analysis. Although sex stratified subgroup analysis could not be performed in our study due to lack of data, based on former 3 studies it can be said that Lys198Asn is associated with ischemic stroke in both sexes.

Our study has several strengths, first of all, this is the first meta‐analysis consisting of a large sample (1,291 cases and 2,513 controls) demonstrating the association between Lys198Asn polymorphism and ischemic stroke. We conducted a comprehensive search, which was not only limited to English literature but also included one paper published in Chinese language. Our analysis included patients of both Asian and Caucasian ethnicity. There was no deviation from Hardy–Weinberg Equilibrium (HWE) in control subjects in all included studies. We performed analysis in both recessive and dominant model. In recessive model, there was no evidence of heterogeneity. However, in dominant model, minimal heterogeneity was seen. Based on the subgroup analysis and the meta‐regression analysis, it was evident that the heterogeneity was due to the difference in the study population and the methodological quality of the included studies. Finally, publication bias was not obvious in our analysis.

There were some limitations in our study. Some studies included in the meta‐analysis had a small sample size and may have provided inconsistent results due to low statistical power. Among the included studies, different methodologies were used for genotyping and the selection of controls. The haplotype analysis, which plays a pivotal role in genetic association studies, was not performed. The gene‐environment interaction and the gene‐gene interaction were not studied in this meta‐analysis. Owing to the above limitations, the results of this meta‐analysis should be interpreted with caution.

## 5 CONCLUSION

Our meta‐analysis suggests a significant association of Lys198Asn polymorphism of EDN1 gene with ischemic stroke. Lys198Asn polymorphism might influence on disease susceptibility and can also be an important marker of risk for ischemic stroke. Large cohort studies with gene–gene and gene–environment interactions in diverse ethnicity should be conducted to reach more reliable conclusion.

## CONFLICT OF INTEREST

The authors declare no conflicts of interest.

## AUTHORS' CONTRIBUTIONS

GN and BKY designed the study. GN and RO carried out literature search, review, and selection. GN and HD carried out the statistical analysis and drafted the manuscript. All authors were involved in revising the manuscript critically for important intellectual content. All authors read and approve the final manuscript.

## ETHICAL APPROVAL

This research does not include human participants.

## INFORMED CONSENT

For this type of study formal consent is not required.

## Data Availability

Data sharing is not applicable to this article as no new data were created or analyzed in this study.
